# Role of Hysteroscopy in the Evaluation of Infertility in a Tertiary Care Hospital in Jammu, India: A Retrospective Study

**DOI:** 10.7759/cureus.113993

**Published:** 2026-08-05

**Authors:** Deepika Sharma, Rohini Jaggi, Shreya Verma

**Affiliations:** 1 Obstetrics and Gynaecology, Government Medical College, Jammu, IND

**Keywords:** endometrial polyp, hysteroscopy, infertility, intrauterine adhesions, submucosal fibroid

## Abstract

Background: Infertility is a serious health issue, affecting approximately 15% of couples of reproductive age worldwide. Abnormal uterine findings have been found in 34%-62% of infertile women.

Methods and materials: This was a retrospective study conducted in the Department of Gynaecology & Obstetrics in Sri Maharaja Gulab Singh (SMGS) Hospital, Jammu, India, for a period of one year, from October 2022 to September 2023. A total of 120 patients with primary/secondary infertility were enrolled in the study. The patients having tubal factors of infertility, abnormal semen analysis, and ovulatory dysfunction were excluded. All these patients were subject to either hysteroscopy alone or hysteroscopy with laparoscopy. Any pathology, such as intrauterine adhesions, polyps, septum, or fibroid, was noted.

Results: Majority (58%) of the patients belonged to the age group of 20-29 years, were nulliparous (68%), and had primary infertility (68%). On hysteroscopy, the majority (52.5%) had a normal cavity, followed by intrauterine adhesions (16.66%), endometrial polyps (14.17%), endometrial hyperplasia (7.5%), submucosal fibroid (5.83%), and bicornuate uterus (3.33%).

Conclusion: In our study, we found that 47.5% of the cases had abnormal findings that were diagnosed by hysteroscopy. It is therefore common to find any pathological finding, especially in patients with unexplained infertility. Moreover, it is quite a simple and short procedure with relatively few complications and must therefore be considered in cases of infertility.

## Introduction

Infertility is a major reproductive health concern affecting individuals and couples worldwide. It is generally recognised when pregnancy does not occur after an appropriate period of regular, unprotected intercourse, although evaluation may begin earlier when age, menstrual disturbance, pelvic disease, or another known risk factor is present. A systematic infertility work-up should assess both partners and include evaluation of ovulation, semen parameters, tubal patency, and the female reproductive tract. Early identification of a correctable cause can reduce delay, guide treatment, and improve the efficiency of fertility care [1]. Uterine factors are an important component of female infertility because implantation requires a receptive endometrial cavity. Endometrial polyps, submucosal leiomyomas, intrauterine adhesions, congenital uterine anomalies, and abnormal endometrial changes may interfere with sperm transport, embryo implantation, or continuation of pregnancy. Some lesions remain asymptomatic and may not be identified during routine pelvic examination. Assessment of the uterine cavity is therefore relevant in women with unexplained infertility, recurrent implantation failure, previous uterine instrumentation, or clinical features suggestive of intrauterine disease [2]. Initial evaluation of the uterine cavity commonly includes transvaginal ultrasonography, hysterosalpingography, or saline-infusion sonography. These investigations are useful and comparatively less invasive, but their diagnostic performance may vary according to the lesion, operator experience, and imaging quality. Hysterosalpingography mainly assesses cavity contour and tubal patency, whereas ultrasonography provides information about the myometrium, endometrium, and adnexa. When findings are inconclusive or a focal lesion is suspected, direct examination of the cavity may be required for definitive diagnosis [3]. Hysteroscopy provides direct visualisation of the cervical canal, uterine cavity, endometrial surface, and tubal ostia. It allows identification of the site, extent, and morphology of intrauterine abnormalities with greater precision than blind procedures. A major advantage is the possibility of combining diagnosis and treatment during the same sitting. Polyps and submucosal fibroids can be resected, adhesions divided, uterine septa corrected, and targeted endometrial sampling performed under direct vision. This "see-and-treat" approach may shorten the diagnostic pathway and avoid repeated procedures [4]. The role of routine hysteroscopy in every infertile woman remains debated when non-invasive imaging shows an apparently normal uterine cavity. Current evidence emphasises careful patient selection based on symptoms, reproductive history, previous imaging, and planned fertility treatment. Office hysteroscopy and smaller-diameter instruments have made evaluation more acceptable and reduced the need for cervical dilatation or general anaesthesia in selected patients. Nevertheless, pain, bleeding, infection, fluid-related complications, and uterine perforation remain recognised concerns, making technique, asepsis, and monitoring essential [5].

The prevalence and spectrum of unsuspected intrauterine abnormalities vary across populations and clinical settings, and evidence from tertiary care centres is valuable because these institutions often manage women with complex, prolonged, or treatment-resistant infertility. Retrospective analyses of routine clinical practice can provide real-world evidence regarding the diagnostic yield of hysteroscopy, the frequency of clinically significant intrauterine lesions, and the proportion of patients who may benefit from simultaneous therapeutic intervention. Such data are important for refining patient selection and optimizing the role of hysteroscopy in infertility evaluation [4,5].

The study aimed to determine the spectrum of hysteroscopic findings and assess their clinical relevance in infertile women attending a tertiary care hospital in Jammu, India. This study is reported in accordance with the Strengthening the Reporting of Observational Studies in Epidemiology (STROBE) guidelines for observational studies.

## Materials and methods

Study design and setting

This retrospective, record-based observational study was conducted in the Department of Obstetrics and Gynaecology, Sri Maharaja Gulab Singh (SMGS) Hospital, Jammu, which is a tertiary care referral hospital of Government Medical College (GMC) Jammu. The study was carried out from 1 February 2026 to 30 June 2026. Medical and operative records of women with primary or secondary infertility who underwent hysteroscopic evaluation during the study period were reviewed.

Study population and sampling technique

The study population consisted of women with primary or secondary infertility who underwent hysteroscopy alone or hysteroscopy combined with laparoscopy during the defined study period. A non-probability, total-enumeration sampling technique was used. All patients who fulfilled the predefined inclusion and exclusion criteria and whose medical records contained adequate clinical, investigative, and operative details were included. After screening the available records, a total of 120 eligible patients were included in the final analysis. As this was a retrospective study, no prospective recruitment, randomisation, or allocation of patients was performed.

Sample size calculation

A formal prospective sample size calculation was not performed because of the retrospective nature of the study. All eligible patients who underwent hysteroscopic evaluation during the one-year study period were included through complete enumeration. Therefore, the final sample size of 120 patients represented the total number of available records that fulfilled the eligibility criteria and contained the complete information required for analysis.

Inclusion criteria

The study included women diagnosed with primary or secondary infertility who had at least one patent fallopian tube, normal semen-analysis findings in their male partners, and documented ovulation occurring either naturally or following ovulation induction. Women who underwent diagnostic hysteroscopy alone or hysteroscopy combined with laparoscopy during the study period were included. Only patients whose medical records contained complete clinical, investigative, and operative information were considered eligible. Thus, the study mainly included patients with unexplained infertility in whom pregnancy had not occurred despite the presence of at least one patent fallopian tube, normal semen parameters, and established ovulation.

Exclusion criteria

Patients with bilateral tubal blockage or any established tubal factor of infertility were excluded from the study. Women whose male partners had an abnormal semen analysis and those with anovulation or established ovulatory dysfunction were also excluded. Patients with incomplete medical or operative records and those in whom the uterine cavity could not be adequately evaluated during hysteroscopy were not included in the final analysis.

Data collection

The medical records, infertility evaluation forms, investigation reports, operative notes, and discharge records of all eligible patients were reviewed. Data were collected using a structured data-recording format. The information recorded included the age of the patient, type and duration of infertility, menstrual history, obstetric history, previous abortions, previous infertility treatment, history of pelvic or uterine surgery, findings of tubal-patency assessment, semen-analysis results, evidence of ovulation, type of procedure performed, and hysteroscopic findings. A detailed clinical history, general physical examination, systemic examination, and local pelvic examination had been performed and documented as part of the routine infertility evaluation of all patients.

Hysteroscopic procedure

All hysteroscopic procedures were performed during the postmenstrual phase of the menstrual cycle to obtain optimal visualisation of the uterine cavity. The procedures were carried out in the operating theatre under general anaesthesia, with the patient placed in the lithotomy position and under complete aseptic precautions. Hysteroscopy was performed using a 2.9-mm, 30-degree telescope manufactured by Karl Storz, along with a 5-mm continuous-flow operative sheath. Normal saline was used as the uterine-distension medium. The posterior vaginal wall was retracted using a Sims speculum, and the anterior lip of the cervix was held with a vulsellum. The hysteroscope was carefully introduced through the cervical canal, and examination of the uterine cavity began at the level of the internal cervical os. The cervical canal, entire uterine cavity, endometrial surface, fundus, lateral uterine walls, and both tubal ostia were systematically inspected. Any intrauterine abnormality, including intrauterine adhesions, endometrial polyps, uterine septum, submucosal fibroids, or other lesions, was identified and documented.

Where clinically indicated and technically feasible, simple operative procedures, such as hysteroscopic adhesiolysis, septal resection, polypectomy, and endometrial curettage, were performed during the same sitting. Combined hysteroscopy and laparoscopy were performed according to the departmental infertility evaluation protocol. Laparoscopy was reserved for women with clinical suspicion of pelvic pathology (e.g., endometriosis, pelvic adhesions, previous pelvic inflammatory disease, chronic pelvic pain, previous pelvic surgery, or abnormal imaging findings) or when comprehensive evaluation of pelvic anatomy and tubal patency was considered clinically indicated. Women without these indications underwent hysteroscopy alone for evaluation of the uterine cavity.

Outcome measures

The primary outcome of the study was to determine the proportion of women with infertility who had abnormal hysteroscopic findings. The secondary outcomes included the distribution and frequency of different intrauterine abnormalities detected during hysteroscopy, the proportion of patients with a normal uterine cavity, the frequency of operative procedures performed during hysteroscopy, and the association of abnormal hysteroscopic findings with the type and duration of infertility and other relevant clinical characteristics.

Potential confounding variables

Potential confounding variables considered in the study included the age of the patient, type and duration of infertility, parity, previous abortions, menstrual abnormalities, history of pelvic or uterine surgery, previous infertility treatment, and the use of ovulation-induction therapy. The effect of major confounding factors was minimised by applying uniform and restrictive eligibility criteria. Only patients with at least one patent fallopian tube, normal semen-analysis findings in the male partner, and documented ovulation were included. Relevant clinical variables were evaluated for their association with abnormal hysteroscopic findings during statistical analysis.

Statistical analysis

The collected data were entered into Microsoft Excel (Microsoft® Corp., Redmond, WA) and analysed using Statistical Product and Service Solutions (SPSS, version 25.0; IBM SPSS Statistics for Windows, Armonk, NY). Categorical variables were presented as frequencies and percentages. Continuous variables were assessed for normality and expressed as mean with standard deviation when normally distributed or as median with interquartile range when non-normally distributed. The chi-square test was used to assess associations between categorical variables, while Fisher's exact test was applied when the expected frequency in any cell was less than five. The independent-samples t-test was used to compare normally distributed continuous variables between two groups, whereas the Mann-Whitney U test was used for non-normally distributed variables. Binary logistic regression analysis was performed, where applicable, to identify factors independently associated with abnormal hysteroscopic findings after adjustment for potential confounding variables. Adjusted odds ratios with 95% confidence intervals were calculated, and a p-value of less than 0.05 was considered statistically significant.

Ethical considerations

The study was conducted after obtaining approval from the Institutional Ethics Committee of GMC Jammu, with approval number IEC/GMCJ/2026/2727, dated 23/01/2026. As the study was retrospective and based on the review of existing hospital records, the requirement for individual written informed consent was waived by the Institutional Ethics Committee, where applicable. The identity and personal information of all patients were kept confidential, and the data were used exclusively for research purposes. The study was conducted in accordance with the ethical principles of the Declaration of Helsinki and relevant institutional guidelines.

## Results

Table 1 presents the demographic and clinical characteristics of the 120 women included in the study. The majority of participants belonged to the 20-29 years age group, comprising 70 (58.33%) patients, followed by 37 (30.83%) women aged 30-39 years and 13 (10.83%) aged 40-49 years, while no participant was aged 50 years or above. The distribution of age groups was statistically significant (Pearson χ² = 94.60, df = 3, p < 0.001), indicating that infertility evaluation was predominantly sought during the younger reproductive age group. With respect to occupation, homemakers constituted the largest proportion of the study population, accounting for 68 (56.67%) patients, whereas 28 (23.33%) were employed in government jobs and 24 (20.00%) were employed in the private sector. The occupational distribution was statistically significant (Pearson χ² = 29.60, df = 2, p < 0.001), demonstrating that the majority of women undergoing infertility evaluation were homemakers. Regarding parity, most participants were nulliparous (P0), representing 82 (68.33%) women, followed by parity one in 35 (29.17%) women and parity two in only three (2.50%) women, while no participant had parity three. The parity distribution showed a highly significant difference (Pearson χ² = 145.27, df = 3, p < 0.001), reflecting that infertility was predominantly observed among women without previous live births. Primary infertility was the most common presentation, affecting 82 (68.33%) women, whereas secondary infertility was observed in 38 (31.67%) women. This distribution was statistically significant (Pearson χ² = 16.13, df = 1, p < 0.001), indicating that primary infertility constituted the major indication for hysteroscopic evaluation in the present study. The duration of infertility ranged from 1 to 15 years, with the majority of women (76; 63.33%) experiencing infertility for one to five years, followed by 39 (32.50%) women with a duration of 6-10 years, and only five (4.17%) women with infertility lasting 11-15 years. None of the participants had infertility for more than 15 years. The distribution according to duration of infertility was statistically significant (Pearson χ² = 124.07, df = 3, p < 0.001), suggesting that most women sought specialist evaluation within the first five years of infertility.

**Table 1:** Demographic and clinical characteristics of the study participants (N = 120) Pearson's chi-square test was applied to the variables. A p-value of less than 0.05 was considered statistically significant.

Variable	Category	Number (n)	Percentage (%)	Pearson χ²	df	p-value
Age (years)	20-29	70	58.33	94.60	3	<0.001
	30-39	37	30.83			
	40-49	13	10.83			
	≥50	0	0.00			
Occupation	Homemaker	68	56.67	29.60	2	<0.001
	Government job	28	23.33			
	Private job	24	20.00			
Parity	P0	82	68.33	145.27	3	<0.001
	P1	35	29.17			
	P2	3	2.50			
	P3	0	0.00			
Type of infertility	Primary infertility	82	68.33	16.13	1	<0.001
	Secondary infertility	38	31.67			
Duration of infertility (years)	1-5	76	63.33	124.07	3	<0.001
	6-10	39	32.50			
	11-15	5	4.17			
	>15	0	0.00			

**Figure 1:**
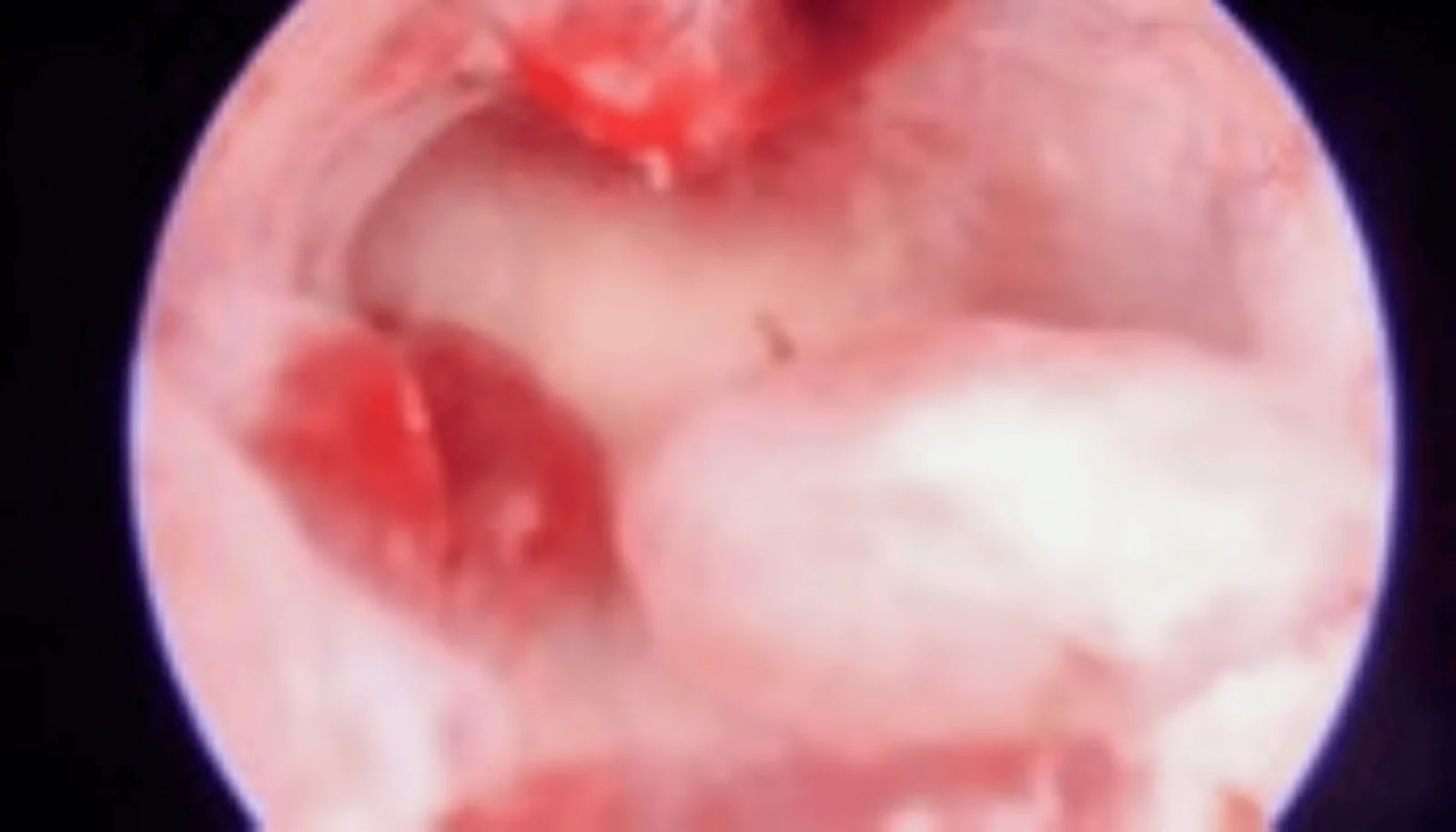
Uterine avity endometrial polyps

Table 2 illustrates the hysteroscopic findings according to the type of infertility. Overall, a normal uterine cavity was observed in 63 (52.50%) women, while 57 (47.50%) had one or more intrauterine abnormalities. Among women with primary infertility, 45 (54.88%) had a normal uterine cavity compared with 18 (47.37%) women with secondary infertility. Intrauterine adhesions were the most frequently detected abnormality, affecting 20 (16.67%) patients overall, including 10 (12.20%) women with primary infertility and 10 (26.32%) with secondary infertility. Endometrial polyps (Figure 1) were identified in 17 (14.17%) women, including 11 (13.41%) with primary infertility and six (15.79%) with secondary infertility. Endometrial hyperplasia was observed in nine (7.50%) patients, submucosal fibroids in seven (5.83%) patients, and bicornuate uterus in four (3.33%) patients, all of which were more frequently encountered among women with primary infertility, except for endometrial hyperplasia. Although abnormal hysteroscopic findings were common in both infertility groups, the overall distribution of hysteroscopic findings between primary and secondary infertility was not statistically significant (Pearson χ² = 9.27, df = 5, p = 0.099).

**Table 2:** Pearson's chi-square test was applied to the variables A p-value of less than 0.05 was considered statistically significant.

Hysteroscopic finding	Primary infertility, n = 82, n (%)	Secondary infertility, n = 38, n (%)	Total, N = 120, n (%)	Pearson χ²	df	p-value
Normal uterine cavity	45 (54.88)	18 (47.37)	63 (52.50)	9.27	5	0.099
Intrauterine adhesions	10 (12.20)	10 (26.32)	20 (16.67)			
Submucosal fibroid	7 (8.54)	0 (0.00)	7 (5.83)			
Bicornuate uterus	4 (4.88)	0 (0.00)	4 (3.33)			
Endometrial hyperplasia	5 (6.10)	4 (10.53)	9 (7.50)			
Endometrial polyp	11 (13.41)	6 (15.79)	17 (14.17)			
Total	82 (100.00)	38 (100.00)	120 (100.00)			

Table 3 summarizes the hysteroscopy-directed operative procedures performed during evaluation. A total of 46 operative procedures were carried out. Polypectomy was the most frequently performed intervention, accounting for 17 (36.96%) procedures, followed by adhesiolysis in 15 (32.61%) patients, curettage in nine (19.57%) patients, and fibroid resection in five (10.87%) patients. The distribution of operative procedures differed significantly (Pearson χ² = 7.91, df = 3, p = 0.048), indicating that hysteroscopic management was predominantly directed toward the treatment of endometrial polyps and intrauterine adhesions.

**Table 3:** Hysteroscopy-directed operative procedures among the study participants Pearson's chi-square test was applied to the variables. A p-value of less than 0.05 was considered statistically significant.

Operative procedure	Number (n)	Percentage (%)	Pearson χ²	df	p-value
Adhesiolysis	15	32.61	7.91	3	0.048
Fibroid resection	5	10.87			
Curettage	9	19.57			
Polypectomy	17	36.96			
Total procedures	46	100.00			

Table 4 depicts the complications encountered following hysteroscopy. A total of 31 complication events were recorded, all of which were minor in nature. Bleeding per vaginum was the most common complication, occurring in 15 (48.39%) events, followed by postoperative pain in 10 (32.26%) events, pyrexia in four (12.90%) events, and nausea with vomiting in two (6.45%) events. Importantly, no cases of uterine perforation, bowel injury, bladder injury, or fainting attacks were reported. The distribution of complication events was statistically significant (Pearson χ² = 58.03, df = 7, p < 0.001), reflecting that minor postoperative complaints predominated, whereas major complications were absent.

**Table 4:** Complications associated with hysteroscopy Pearson's chi-square test was used to determine statistical significance. A p-value of less than 0.05 was considered statistically significant.

Complication	Number (n)	Percentage (%)	Pearson χ²	df	p-value
Uterine perforation	0	0.00	58.03	7	<0.001
Postoperative pain	10	32.26			
Bleeding per vaginum	15	48.39			
Pyrexia	4	12.90			
Nausea and vomiting	2	6.45			
Fainting attack	0	0.00			
Bowel injury	0	0.00			
Bladder injury	0	0.00			
Total complication events	31	100.00			

## Discussion

In the present study, most women were aged 20-29 years, accounting for 70 (58.33%) of the 120 participants, while 37 (30.83%) were aged 30-39 years, and 13 (10.83%) were aged 40-49 years. Primary infertility was observed in 82 (68.33%) women, compared with secondary infertility in 38 (31.67%). Moreover, 82 (68.33%) women were nulliparous, 68 (56.67%) were homemakers, and 76 (63.33%) had experienced infertility for one to five years. These findings indicate that most patients presented for infertility evaluation during the younger reproductive years and relatively early in the course of infertility. Chanu et al. reported a similar predominance of primary infertility among 151 women undergoing diagnostic hysterolaparoscopy, with 88 (58.28%) having primary infertility and 63 (41.72%) having secondary infertility. They also observed that women with primary infertility were predominantly in the 25-30-year age group, whereas women with secondary infertility were generally older. The higher proportion of primary infertility in both studies supports the observation that women who have never conceived constitute a major proportion of patients referred to tertiary infertility centres [6].

Hysteroscopy revealed a normal uterine cavity in 63 (52.50%) women in the present study, whereas 57 (47.50%) had an intrauterine abnormality. Abnormal findings were detected in 37 of 82 (45.12%) women with primary infertility and 20 of 38 (52.63%) women with secondary infertility. Although abnormalities were numerically more frequent in secondary infertility, the overall distribution of hysteroscopic findings did not differ significantly between the two groups (χ² = 9.27, df = 5, p = 0.099). The relatively small number of women with secondary infertility may have limited the statistical power to detect a modest but clinically meaningful difference between the two groups. Therefore, the absence of statistical significance should not be interpreted as evidence of equivalence between primary and secondary infertility with respect to intrauterine pathology. Larger multicentre studies with adequate sample sizes are needed to clarify whether this observed difference represents a true clinical association.

Pansky et al., in a retrospective study of 221 infertile women, detected an abnormal uterine cavity in 30.00% of patients, which was lower than the 47.50% observed in the present study. However, similar to the present findings, they found no statistically significant difference in the overall frequency of intrauterine abnormalities between women with primary and secondary infertility. The higher abnormality rate in the present study may be related to differences in referral patterns, patient selection, previous reproductive history, and the tertiary-care setting [7].

Intrauterine adhesions were the most common abnormality in the present study, occurring in 20 (16.67%) women, followed by endometrial polyps in 17 (14.17%), endometrial hyperplasia in nine (7.50%), submucosal fibroids in seven (5.83%), and bicornuate uterus in four (3.33%). Adhesions were more frequent in secondary infertility than in primary infertility, affecting 10 of 38 (26.32%) and 10 of 82 (12.20%) women, respectively. Fatemi et al. prospectively evaluated 678 asymptomatic infertile women with normal transvaginal ultrasonography before their first in vitro fertilization (IVF) or intracytoplasmic sperm injection (ICSI) cycle and detected intrauterine abnormalities in only 11.00% of patients. They identified endometrial polyps in 6.00% (41) of women, intrauterine adhesions in approximately 2.00%, uterine septa in 2.00%, and submucosal myomas in 1.00% (6). Thus, the frequencies of polyps, adhesions, and submucosal fibroids were higher in the present study. This difference may be explained by the inclusion of a broader tertiary-care infertility population in the present study, whereas Fatemi et al. included asymptomatic women with normal ultrasonographic findings who were preparing for their first assisted-reproduction cycle [8].

A total of 46 hysteroscopy-directed operative procedures were performed in the present study, confirming the therapeutic as well as diagnostic role of hysteroscopy. Polypectomy was the most frequent procedure, accounting for 17 (36.96%) of all interventions and 14.17% of the total study population. This was followed by adhesiolysis in 15 (32.61%) procedures, representing 12.50% of the total cohort, curettage in nine (19.57%) procedures, representing 7.50% of the cohort, and fibroid resection in five (10.87%) procedures, representing 4.17% of the cohort. Citu et al. similarly reported that operative hysteroscopy was performed in all patients with detected uterine abnormalities. Among 198 infertile women, they performed polypectomy in 78 (39.39%), resection of uterine synechiae in 21 (10.61%), hysteroscopic myomectomy in nine (4.55%), metroplasty in seven (3.54%), and endometrial resection in eight (4.04%). Polypectomy was therefore the most frequently performed intervention in both studies, although adhesiolysis constituted a comparatively larger proportion of procedures in the present study. These findings demonstrate that hysteroscopy permits the immediate treatment of correctable intrauterine lesions during the same operative session [9].

A total of 31 minor postoperative events were recorded in the present study. Bleeding per vaginum was reported in 15 women, representing 12.50% of the total cohort and 48.39% of all recorded events. Postoperative pain occurred in 10 (8.33%) women and constituted 32.26% of the events, while pyrexia occurred in four (3.33%) women and nausea and vomiting in two (1.67%). No uterine perforation, bowel injury, bladder injury, or fainting episode was recorded. Jansen et al., in a prospective multicentre study of 13,600 hysteroscopic procedures, reported 38 complications, giving an overall complication rate of 0.28%. The complication rate was 0.13% for diagnostic hysteroscopy and 0.95% for operative hysteroscopy, while uterine perforation occurred at a rate of 0.76% among operative procedures. Procedure-specific complication rates were 4.48% for adhesiolysis, 0.75% for myomectomy, and 0.38% for polypectomy. Direct comparison with the present study should be made cautiously because Jansen et al. defined complications as unexpected events requiring additional intervention [10], whereas the present study also recorded minor symptoms, such as pain, limited bleeding, pyrexia, and nausea. Nevertheless, the absence of uterine perforation and visceral injury in the present study supports the safety of hysteroscopy when performed under appropriate operative conditions. The statistically significant chi-square value for the distribution of complications reflects variation among the types of recorded events and should not itself be interpreted as a measure of procedural safety [10].

Falloposcopy may provide additional information regarding intraluminal tubal pathology in selected patients with unexplained infertility [10]. However, because of its limited availability, technical complexity, and restricted use in routine clinical practice, it was beyond the scope of the present study.

Strengths and limitations

It reflects real-world clinical practice in a tertiary care referral centre and included all eligible women who underwent hysteroscopic evaluation during the study period through complete enumeration, thereby minimizing sampling bias within the study setting. Uniform inclusion criteria ensured that all participants had at least one patent fallopian tube, documented ovulation, and normal semen parameters in their partners, allowing the study to specifically evaluate the contribution of uterine cavity abnormalities in women with otherwise unexplained infertility. Furthermore, hysteroscopic findings were obtained through direct visualization of the uterine cavity, which is considered the reference standard for detecting intrauterine pathology.

However, the study also has a few limitations. First, its retrospective, single-centre design limits the generalizability of the findings to other healthcare settings and populations. Second, the relatively modest sample size, particularly the smaller number of women with secondary infertility, may have reduced the statistical power to detect differences between primary and secondary infertility groups. Third, standardized classification systems, such as the American Society for Reproductive Medicine (ASRM) classification for intrauterine adhesions and the International Federation of Gynecology and Obstetrics (FIGO) classification for submucosal leiomyomas, were not consistently documented in the operative records because of the retrospective nature of the study. Consequently, lesion severity could not be analysed. Finally, the study evaluated hysteroscopic findings at the time of diagnosis but did not include follow-up data on reproductive outcomes after hysteroscopic intervention, such as spontaneous conception rates, assisted reproductive technology outcomes, or live birth rates. Prospective multicentre studies with larger sample sizes, standardized reporting of intrauterine lesions, and long-term reproductive follow-up are warranted to further define the role of hysteroscopy in infertility evaluation and management.

## Conclusions

Hysteroscopy is a valuable diagnostic and therapeutic tool in the evaluation of women with infertility. Intrauterine abnormalities were detected in nearly half of the patients, with adhesions and endometrial polyps being the most common findings. The procedure also allowed immediate operative correction of several abnormalities with only minor complications. Thus, hysteroscopy can be safely incorporated into the infertility work-up, particularly in selected women with unexplained infertility.
